# Mutation Inactivation of Nijmegen Breakage Syndrome Gene (*NBS1*) in Hepatocellular Carcinoma and Intrahepatic Cholangiocarcinoma

**DOI:** 10.1371/journal.pone.0082426

**Published:** 2013-12-13

**Authors:** Yan Wang, Yu Hong, Man Li, Jiang Long, Yan-Ping Zhao, Jun-Xia Zhang, Qian Li, Hong You, Wei-Min Tong, Ji-Dong Jia, Jian Huang

**Affiliations:** 1 Liver Research Center, Beijing Friendship Hospital, Capital Medical University, Beijing, China; 2 Department of Pathology, Institute of Basic Medical Sciences, Chinese Academy of Medical Sciences & Peking Union Medical College, Beijing, China; 3 Minimally Invasive Hepatobiliary Cancer Center, Beijing You-An Hospital, Capital Medical University, Beijing, China; 4 Department of Hepatology, Tianjin Infectious Disease Specialty Hospital, Tianjin, China; 5 Department of Preventive Medicine, Mt. Sinai School of Medicine, New York, New York, United States of America; Medical University Graz, Austria

## Abstract

Nijmegen breakage syndrome (NBS) with *NBS1* germ-line mutation is a human autosomal recessive disease characterized by genomic instability and enhanced cancer predisposition. The *NBS1* gene codes for a protein, Nbs1(p95/Nibrin), involved in the processing/repair of DNA double-strand breaks. Hepatocellular carcinoma (HCC) is a complex and heterogeneous tumor with several genomic alterations. Recent studies have shown that heterozygous *NBS1* mice exhibited a higher incidence of HCC than did wild-type mice. The objective of the present study is to assess whether *NBS1* mutations play a role in the pathogenesis of human primary liver cancer, including HBV-associated HCC and intrahepatic cholangiocarcinoma (ICC). Eight missense *NBS1* mutations were identified in six of 64 (9.4%) HCCs and two of 18 (11.1%) ICCs, whereas only one synonymous mutation was found in 89 control cases of cirrhosis and chronic hepatitis B. Analysis of the functional consequences of the identified *NBS1* mutations in Mre11-binding domain showed loss of nuclear localization of Nbs1 partner Mre11, one of the hallmarks for Nbs1 deficiency, in one HCC and two ICCs with *NBS1* mutations. Moreover, seven of the eight tumors with *NBS1* mutations had at least one genetic alteration in the *TP53* pathway, including *TP53* mutation, *MDM2* amplification, *p14ARF* homozygous deletion and promoter methylation, implying a synergistic effect of Nbs1 disruption and p53 inactivation. Our findings provide novel insight on the molecular pathogenesis of primary liver cancer characterized by mutation inactivation of *NBS1*, a DNA repair associated gene.

## Introduction

Primary liver cancer is the second most common cancer in Asia and the fourth most common cancer in Africa. In 2002, the global number of new cases in males was estimated to be 442 119; there were 416 882 deaths, 94% of which occurred in the first year after diagnosis [1,2]. Primary liver cancer comprises mainly hepatocellular carcinoma (HCC; about 90%) and intrahepatic cholangiocarcinoma (ICC; 5–15%) [1,2,3]. Hepatitis B virus (HBV) infection is widely recognized as an extremely high risk factor for HCC and ICC progression [3,4].

It is known that multiple risk factors, including HBV and hepatitis C virus infection, aflatoxin contamination, alcohol abuse, ionizing radiation and human metabolic products toxic to the human genome, can cause DNA damage such as double-strand breaks (DSBs), single-strand breaks and point mutation in hepatocytes [5]. DNA repair is essential when DNA damage occurs, and defects in this process may lead to fatal conditions such as chromosomal instability syndromes and cancer [5,6]. Notably, HBV DNA was found in the genome of almost all cases of HBV-associated HCC, and the efficiency of integration is enhanced when DSBs or oxidative DNA damage occurs [7,8]. Thus, accumulation of damaged DNA due to impaired DNA damage repair can become an important molecular mechanism in the carcinogenesis of HCC, especially HBV-associated HCC.

Nijmegen breakage syndrome (NBS), caused by a germline mutation (657del5) in the *NBS1* gene, is an autosomal recessive chromosomal instability syndrome characterized by predisposition to cancer, especially leukemia and lymphoma [9]. The product of the *NBS1* gene, Nbs1(p95/Nibrin), is a component of the Mre11/Rad50/Nbs1 (MRN) complex, which is localized in the nucleus and acts as a DNA DSBs sensor and functions in the cell cycle checkpoint in response to DNA damage [6]. Following DSBs in DNA, Nbs1 interacts with phosphorylated H2AX (γ-H2AX) and is responsible for nuclear translocation of the Mre11/Rad50 repair complex to sites of DNA damage where it senses DNA strand breaks and activates ataxia telangiectasia mutated (ATM) [10]，which is central to the DSBs response in mammalian cells. In addition, Nbs1 is phosphorylated by ATM, activating downstream molecules including p53, BRCA1 and Chk2 to control cell cycle progression [6,10]. Thus, Nbs1 plays crucial roles in ATM-dependent DNA damage responses and the maintenance of genome stability.

Accumulated evidence suggests a role of Nbs1 in tumorigenesis. In addition to lymphoma and leukemia in NBS patients, *NBS1* mutations have been found in sporadic cancers, including breast cancer [11], colorectal cancer [12], medulloblastoma [13], primary glioblastomas [14], lymphoid malignancies and acute lymphoblastic leukemia [15]. Moreover, there may be functional interactions between Nbs1 and p53 [16,17], and mutational inactivation of the *NBS1* gene in tumors is associated with *TP53* mutations [13,14], suggesting a synergistic effect of Nbs1 with p53 in the development of cancer.

Notably, it was reported recently that heterozygous *NBS1* mice exhibited a higher incidence of HCC than did wild-type mice (http://escholarship.org/uc/item/16t4k4cd). Other research has shown that mice heterozygous for NBN (the murine homolog of *NBS1*) developed HCC in addition to lymphomas [18]. It is noteworthy that in almost all HBV-associated HCC cases, HBV DNA integrates into the host genome with an enhanced efficiency of integration when DSBs occurs. This has raised the question of whether Nbs1 plays a role in the pathogenesis of primary liver cancer, especially HBV-associated HCC.

To explore the role of Nbs1 in liver carcinogenesis, we screened and analyzed mutations in the *NBS1* gene and genetic alterations in the *TP53* pathway. Moreover, we evaluated the functional consequences of the identified *NBS1* mutations through the analysis of Nbs1 phosphorylation and nuclear localization of Nbs1 partner Mre11.

## Materials and Methods

### Ethics Statement

The study protocol was approved by the Clinical Research Ethics Committee of Beijing Friendship Hospital, Capital Medical University. All participants provided their written informed consent to participate in this study, and the ethics committees approved this consent procedure. All data on DNA sequencing of the identified NBS1 mutations have been deposited in GenBank with accession numbers of JN390965 and KF147842-KF147848.

### Tissue samples and cell lines

Eighty-two primary liver cancer patients, comprising 67 men (81.7%) and 15 women (18.3%), aged 27–78 years with a median age of 53.9 years, were randomly enrolled in this study. The patients underwent surgical treatment at the Liver Research Center, Beijing Friendship Hospital, Capital Medical University, Department of Hepatology, Tianjin Infectious Disease Specialty Hospital and the Minimally Invasive Hepatobiliary Cancer Center, Beijing You-An Hospital between January, 2005 and December, 2010. Seventy-six tumors were fixed in buffered formalin and embedded in paraffin; 17 frozen tumors were paired with adjacent non-tumor tissues. Another six pairs of frozen tumors not prepared as paraffin-embedded sections were also included in the study.

The patients were diagnosed as follows: (1) HCC or ICC; (2) with or without HBV infection, determined by positivity or negativity for hepatitis B surface antigen; (3) tumor stage 1 (corresponding to TNM stage I, T1N0M0, as classified by the Union for International Cancer Control) or >1; and (4) well, moderately or poorly differentiated tumor, classified according to the World Health Organization Classification of Tumors of the Digestive System (Table 1) [1,2]. 

**Table 1:** Summary of clinicopathological characteristics of patients with HCC or ICC.

Characteristic	Tumor cases		
	HCC	ICC	PLC*
Case number	64	18	82
Mean age (range)	54.2 (27–78)	52.6 (32–75)	53.9 (27–78)
Gender			
Male	53 (82.8%)	14 (77.8%)	67 (81.7%)
Female	11 (17.2%)	4 (22.2%)	15 (18.3%)
Tumor stage			
Stage 1	38 (59.4%)	4 (22.2%)	42 (51.2%)
Stage 1	26 (40.6%)	14 (77.8%)	40 (48.8%)
HBV infection			
Positive	52 (81.3%)	12 (66.7%)	64 (78.1%)
Negative	12 (18.7%)	6 (33.3%)	18 (21.9%)
Differentiation			
Well	10 (15.6%)	1 (5.5%)	11 (13.4%)
Moderate	38 (59.4%)	13 (72.2%)	51 (62.3%)
Poor	16 (25.0%)	4 (22.3%)	20 (24.3%)
*NBS1* mutation			
Yes	6 (9.4%)	2 (11.1%)	8 (9.8%)
No	58 (90.6%)	16 (88.9%)	74 (90.2%)

^*^ PLC: primary liver cancer.

Eighty-nine biopsies of tissue from patients with HBV-associated cirrhosis or chronic hepatitis B were used as controls for the screening for *NBS1* mutations, comprising 53 cases of HBV-associated cirrhosis and 36 cases of HBV-associated chronic hepatitis B diagnosed at the Beijing Friendship Hospital and Tianjin Infectious Disease Specialty Hospital.

HCC cell lines (HepG2 and Hep-3B) and ICC cell line (HCCC-9810) were purchased from the National Platform of Experimental Cell Resource for Sci-Tech (Beijing, China). HCC cell line (HepG2.2.15) was provided by Beijing Institute of Radiation Medicine, originated from the Mount Sinai Medical Center in New York.

### Single-stranded conformational polymorphism (SSCP) analysis and direct DNA sequencing for *NBS1* mutations

SSCP analysis was conduct to prescreen for mutations in all 16 exons of the *NBS1* gene. Extraction of genomic DNA from paraffin sections and PCR-SSCP analysis for *NBS1* were conducted as described previously [13,14,19]. Mutations identified in paraffin sections were verified by sequencing a second product of amplification on both strands and were further validated using paired frozen tissue.

### Detection of genetic alterations in the TP53 pathway

Prescreening for mutations in exons 5–8 of the *TP53* gene by PCR-SSCP analysis followed by direct DNA sequencing was conducted as described previously [13]. Differential PCR for *p14ARF* homozygous deletion and *MDM2* amplification, as well as methylation-specific PCR for *p14ARF* promoter methylation, were conducted as described previously [13].

### Immunohistochemistry (IHC) and Immunofluorescence (IF)

Sections (4 µm thick) were cut for IHC. After deparaffinization of the slides, endogenous peroxidase activity was blocked with 0.3% H_2_O_2_ in methanol for 30 min. Antigen retrieval was performed in antigen unmasking solution (Vector H-3300) with microwaving for 15 min, keeping the solution boiling, followed by treatment with 5% skimmed milk in phosphate buffered saline (PBS)-0.1% bovine serum albumin for at least 1 h at room temperature to block nonspecific staining.

Immunohistochemical staining was performed using antibodies against Nbs1 (1:1000; Abcam), p-Nbs1 (1:50; Novus), and Mre11 (1:4000; Abcam) at 4°C overnight. Secondary antibody (Vector MP-7401) was used at 37°C for 1 h, and visualization of antigen–antibody reactions was achieved with 3,3'-diaminobenzidine (Vector SK-4100). Tissue structures were visualized by counterstaining with hematoxylin.

For IF, five micrometer thick frozen sections were cut using Cryocut (Leica Microsystems, Wetzlar, Germany) and fixed in ice-cold acetone-methanol (1:1) for 30 min on ice. The slides were then incubated with rabbit anti-Mre11 (Novus, 1:4000; Abcam) in Tris buffered saline with Tween containing 5% non-fat dried milk at 4°C overnight. After three washes in PBS, the primary antibody was detected with the corresponding fluorescein isothiocyanate-conjugated anti-IgG (Molecular Probes, Eugene, OR) at 37°C for 20 min. Sections were examined under a Zeiss Axioskop fluorescence microscope equipped with a charge coupled device imaging system (IPLab Spectrum; Signal Analytics Corp., Vienna, VA).

### Western blot

Frozen tissues or cell cultures were lysed and clarified by centrifugation. The protein concentration was determined using a BCA kit (Pierce, Rockford, IL). Eighty micrograms of each protein extract was loaded onto 10% SDS-PAGE gel and transferred to nitrocellulose membrane. The membrane was probed with primary antibody against Nbs1 (Abcam, Cambridge, MA), phosphorylated Nbs1 (p-Nbs1, 1:1000; phospho-Ser343, Novus, Littleton, CO), with β-actin (Sigma, St. Louis, MO) as the loading control. The membrane was then incubated with species-specific secondary horseradish peroxidase-conjugated antibodies (Sigma). Protein bands were revealed by chemiluminescence (Pierce) and detected on X-ray films (Kodak, Rochester, NY).

### Statistical analysis

The chi-square test (for expected values > 5) and Fisher’s exact test (for expected values ≤ 5) were used to determine molecular associations using SAS v9.2 software (SAS Institute, Inc., Cary, NC). *P* < 0.05 was considered statistically significant for all tests.

## Results

### High rate of *NBS1* mutations identified in HCC and ICC but not in cirrhosis or chronic hepatitis B

**Figure 1:**
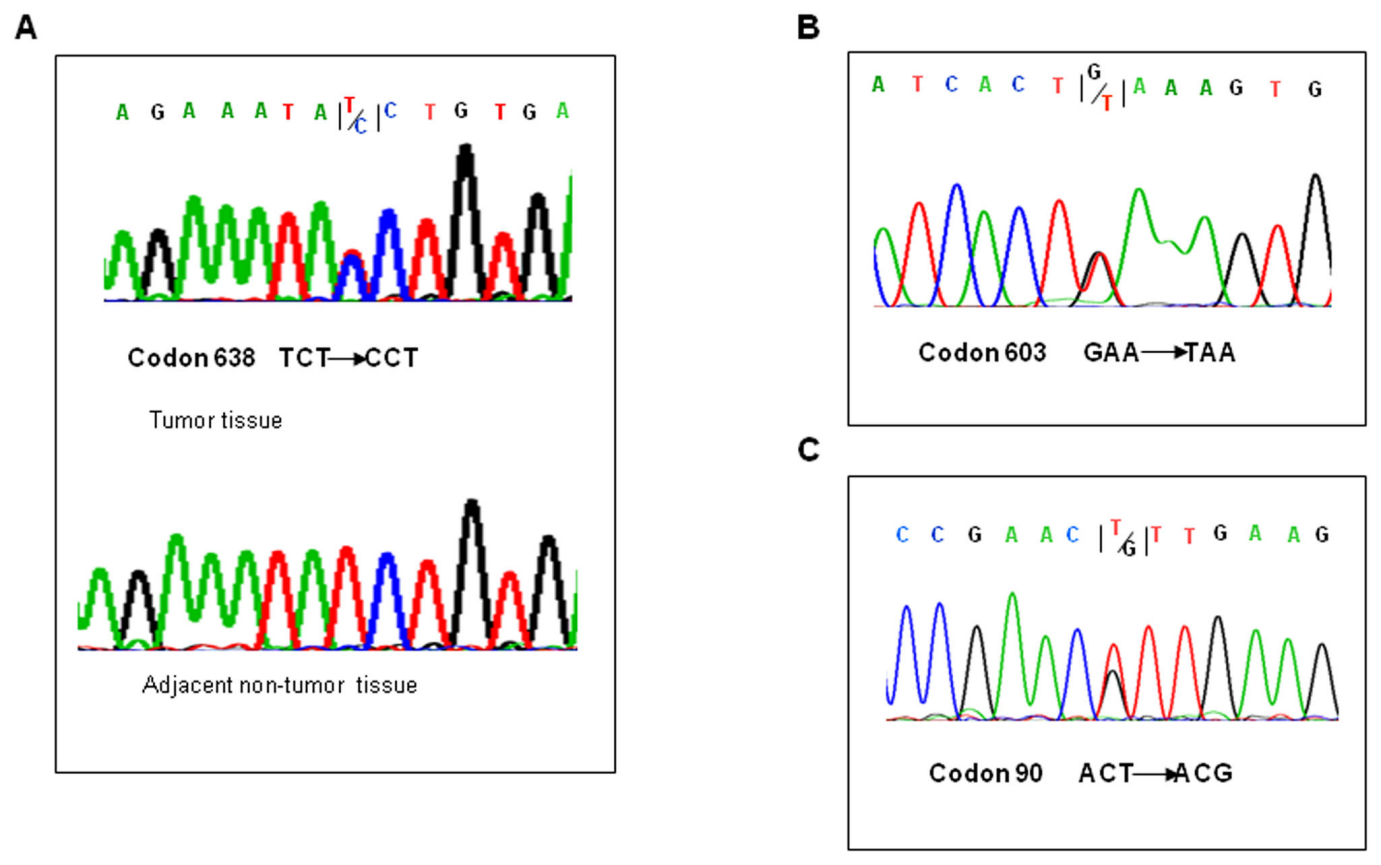
Representative DNA sequencing of *NBS1* mutations in HCC and ICC. (A) Missense NBS1 mutation at codon 638 (TCT→CCT, Ser→Pro) was identified in a case of ICC, but not in the adjacent non-tumorous tissue, indicating that the mutation was somatic. (B) Missense NBS1 mutation at codon 603 (TTC→TTA, Phe→Leu) in a case of HCC. The reverse complementary sequence is shown. (C) Synonymous NBS1 mutation at codon 90 (ACT→ACG, Thr→Thr) in a case of HBV-associated cirrhosis.

**Figure 2:**
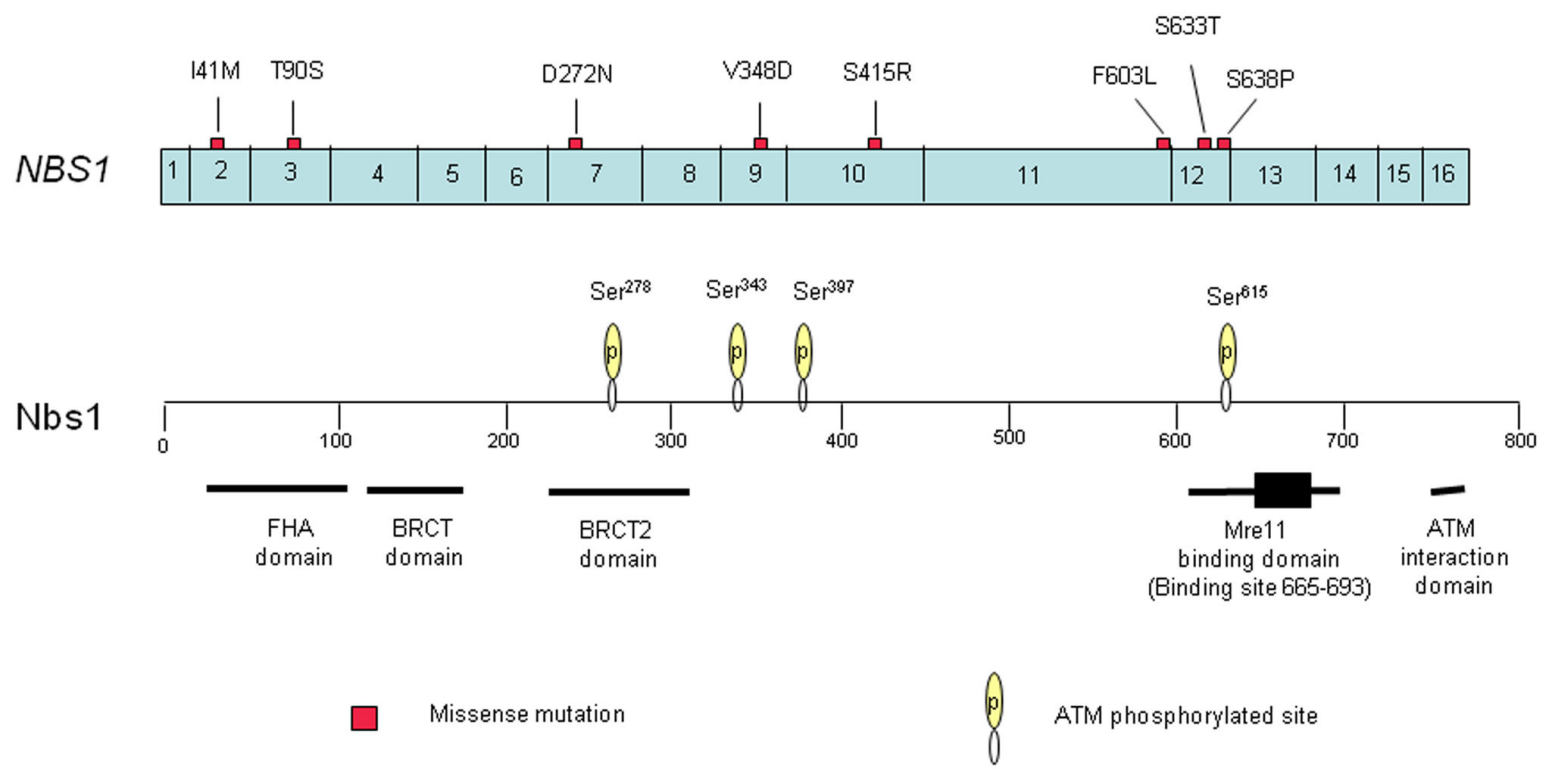
Distribution and type of *NBS1* mutations in HCC and ICC. Mutations are located preferentially in exon 11/12 (Mre11-binding domain), but also in or close to other functional domains (FHA domain; second BRCT (BRCT2) domain; ATM phosphorylated sites, Ser278/Ser343/Ser397/Ser615).

Eighty-two cases of primary liver cancer, including 64 cases of HCC and 18 cases of ICC, were screened for *NBS1* mutations. SSCP followed by direct sequencing of all 16 exons of *NBS1* revealed eight missense *NBS1* mutations in eight of the 82 (11.0%) cases of primary liver cancer (Table 2, Figure 1A-B), distributed among exons 2, 3, 7, 9, 10,11 and 12 (Figure 2). In five cases with *NBS1* mutations (cases 217, 375, 383, 425 and 478), adjacent non-tumor tissue was available. No *NBS1* mutations were found in DNA extracted from non-tumor tissue, indicating that the *NBS1* mutations in the tumors were somatic (Figure 1A). 

**Table 2:** *NBS1* miscoding mutations identified in HCC and ICC.

Patient ID	Clinical parameters^*^	Age/sex	*NBS1* mutation	Alteration in *TP53* pathway
217	HCC/HBV(+)/Stage1/md	50/M	Codon 41, ATC→ATG, Ile→Met	*TP53* mutation, P301L
				*p14ARF* deletion
375	HCC/HBV(+)/Stage1/wd	54/M	Codon 633, TCA→ACA, Ser→Thr	*p14ARF* promoter methylation
478	HCC/HBV(–)/Stage1/md	67/M	Codon 272, GAT→AAT, Asp→Asn	*p14ARF* promoter methylation
383	HCC/HBV(+)/Stage1/md	48/M	Codon 348, GTT→GAT, Val→Asp	*TP53* mutation, Y220C
354	HCC/HBV(+)/Stage1/wd	46/M	Codon 415, AGT→AGA, Ser→Arg	*MDM2* amplification
339	HCC/HBV(+)/Stage1/md	42/M	Codon 603, TTC→TTA, Phe→Leu	–
425	ICC/HBV(–)/Stage1/md	52/F	Codon 638, TCT→CCT, Ser→Pro	*TP53* mutation, Q192H
				*p14ARF* promoter methylation
362	ICC/HBV(+)/Stage1/wd	38/M	Codon 90, ACT→TCT, Thr→Ser	*p14ARF* promoter methylation

^*^ HBV(+)/HBV(–): with/without hepatitis B virus infection; Stage 1/Stage1: tumor stage 1/>1; wd/md/pd: well/moderately/poorly differentiated.

Of the eight *NBS1* mutations, six were identified in 64 HCC cases, with two in 18 ICC cases (*P*=1.0000). No significant differences in the occurrence of *NBS1* mutations were observed between different tumor stages, degrees of differentiation or the presence or absence of HBV infection in HCC cases (Table S1).No mutation was identified in poor differentiated tumors (Table 2, Table S1). 

Common *NBS1* single nucleotide polymorphisms (SNPs) in tumor cases were identified at similar frequencies as that in control cases of cirrhosis or chronic hepatitis B, except for SNP D399D, which was significantly more frequent in tumor cases [18] .Two rare SNPs (N716D and E564K) and three splicing variants (IVS12–15 A→C, IVS 6+57 T→A and IVS 5+115 A→G) in *NBS1* gene were identified in five HCC cases respectively. 

In contrast, no miscoding *NBS1* mutations or rare SNPs were identified in any of the 89 control cases of cirrhosis or chronic hepatitis B, with the exception of one synonymous *NBS1* mutation identified in a case of HBV-associated cirrhosis (ACT→ACG, T90T; Figure 1C). These results suggest that the rate of *NBS1* mutation is significantly higher in primary liver cancer than in cirrhosis or chronic hepatitis B (*P* =0.0023).

### Mutation in Mre11-binding domain of *NBS1* may impair nuclear localization of the Nbs1 partner Mre11

The potential effects of the eight *NBS1* missense mutations on Nbs1 protein function were investigated using the Polymorphism Phenotyping (PolyPhen-2) algorithm, which is used to predict the possible functional impact of an amino acid substitution [20]. Five missense *NBS1* mutations (I41M, D272N, V348D, S633T and S638P) were predicted to be damaging to Nbs1 function.

**Figure 3:**
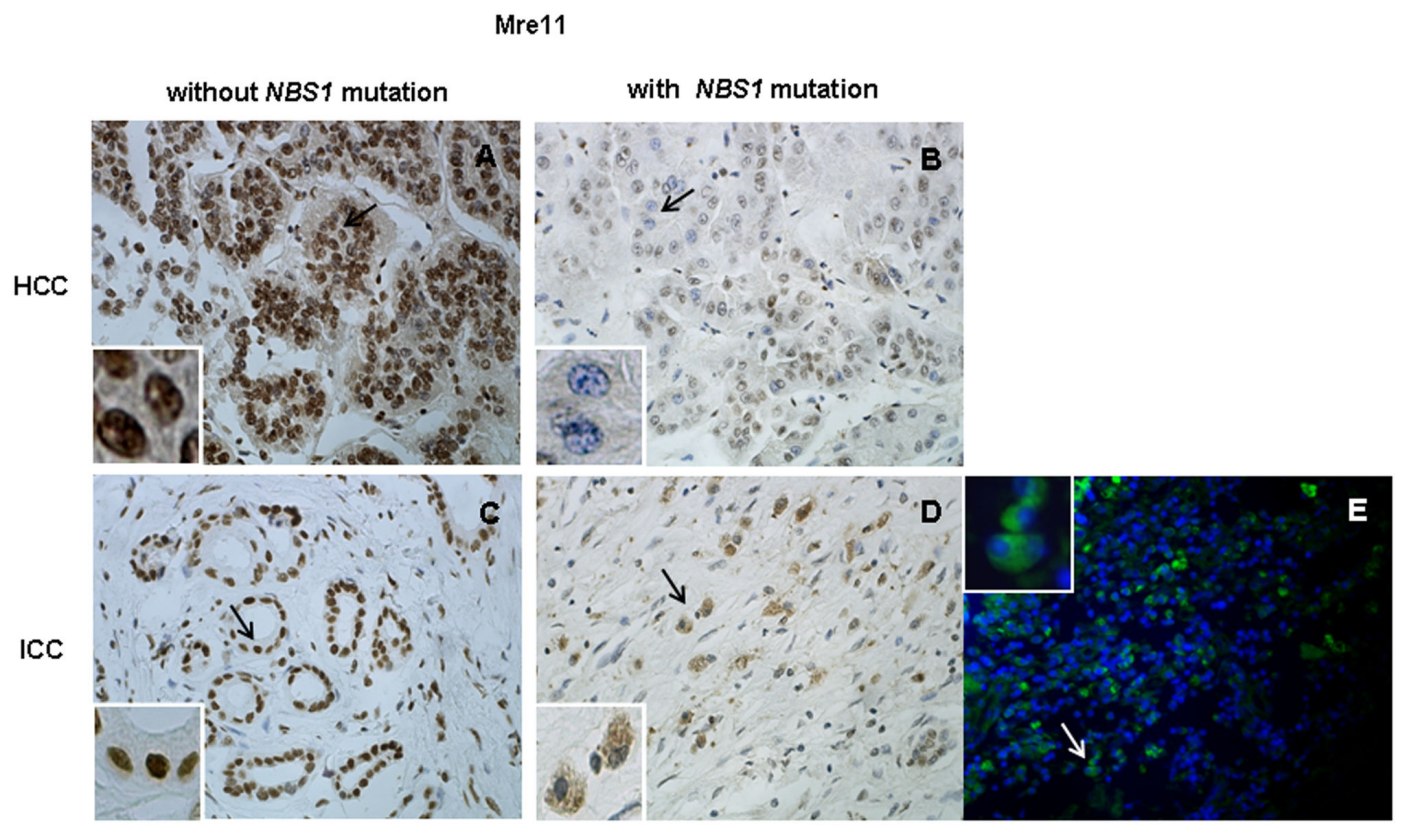
Mre11 nuclear staining in tumor cells with or without *NBS1* mutations. Upper panel: IHC of a pair of HCC cases, (A) without NBS1 mutation (case 374) and (B) with NBS1 mutation S633T (case 375). Bottom panel: IHC of a pair of ICC cases, (C) without NBS1 mutation (case 382) and (D) with NBS1 mutation S638P (case 425); immunofluorescence staining of case 425 for which frozen tissue was available (E). The black/white arrows indicate tumor cells with nuclear or cytoplasmic staining of Mre11. Loss of Mre11 nuclear staining was observed in tumor cells from cases 375 and 425 with NBS1 mutations in the Mre11-binding domain (S633T and S638P). Original magnification: ×40.

Because three of the eight missense mutations located in the binding domain of Nbs1, we examined Mre11 nuclear staining by performing IHC and IF assays (when frozen tissue was available) on all tumors with *NBS1* mutations to determine whether the *NBS1* mutations have functional consequences on the binding of Nbs1 to Mre11; 10 HCC and 10 ICC cases without *NBS1* mutations served as controls. Disruption of the Mre11 binding domain of Nbs1 may lead to loss of Mre11 nuclear localization and increased staining for Mre11 in the cytoplasm [21]. Strong Mre11 nuclear staining was observed in all tumors without *NBS1* mutations. Down-regulation and/or loss of nuclear localization of Mre11 with cytoplasmic Mre11 staining was observed in three of the eight tumors with *NBS1* mutations: case 425 with mutation S638P, case 375 with mutation S633T and case 362 with mutation T90S (Figure 3A–E). S638P and S633T are in the Mre11 binding domain (601–700) of Nbs1 and may impair binding of Mre11 to Nbs1, consistent with the previous report described above.

We next analyzed the impact of *NBS1* mutations on Nbs1 phosphorylation by western blot and IHC analysis. Altered Nbs1 phosphorylation was not observed in any of the tumors with *NBS1* mutation (Figure S1).

### *NBS1* mutations frequently accompanied with genetic alterations in the *TP53* pathway

**Figure 4:**
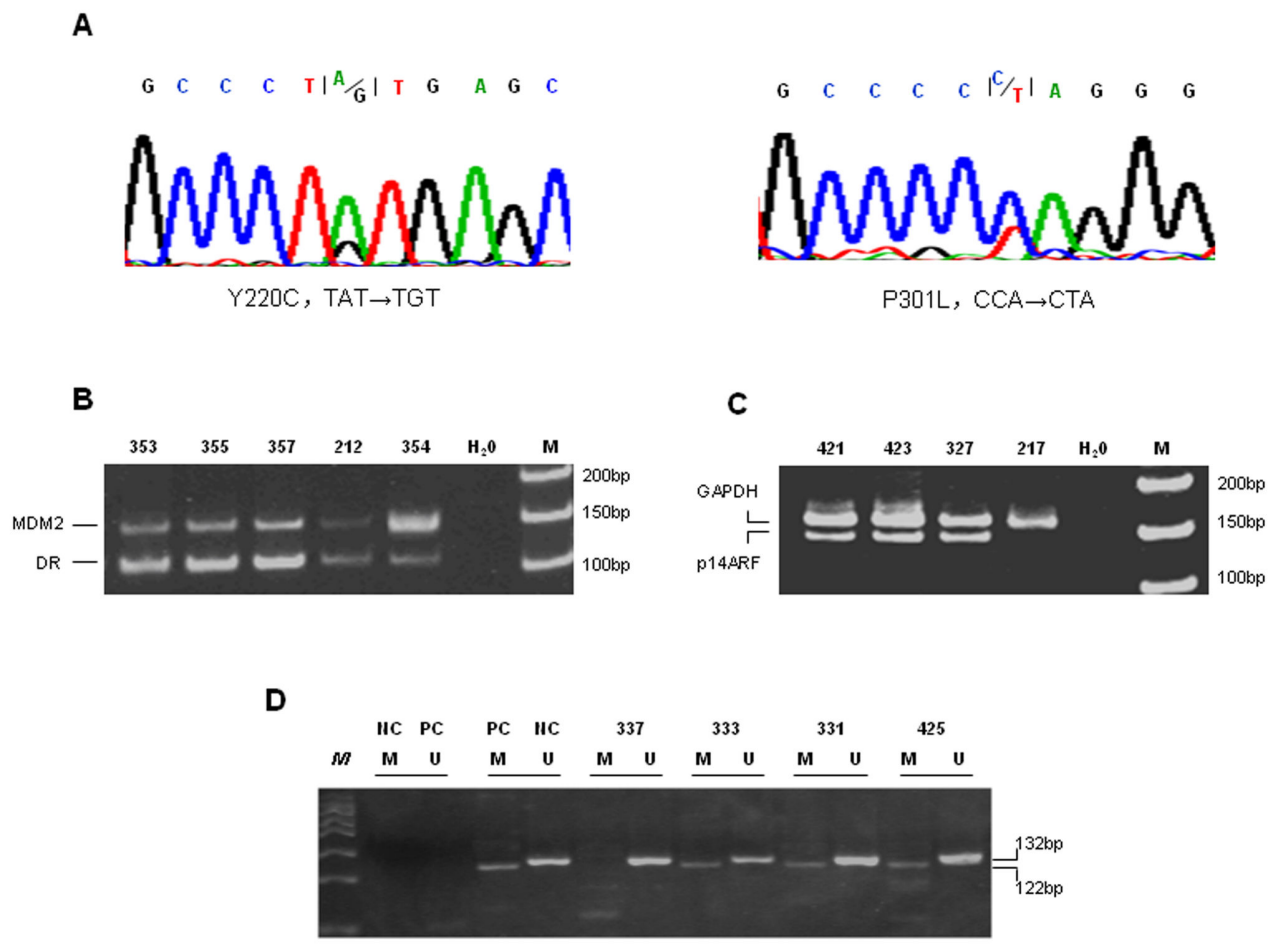
Genetic alterations in the *TP53* pathway in representative tumor cases. (A) Representative DNA sequencing of *TP53* mutations: *TP53* mutation Y220C in case 383 with NBS1 mutation V348D, *TP53* mutation P301L in case 217 with *NBS1* mutation I41M. (B) *MDM2* amplification in case 354 with *NBS1* mutation S415R. The dopamine receptor (DR) sequence was used as the reference. (C) *p14ARF* homozygous deletion in case 217 with *NBS1* mutation I41M. The *GAPDH* sequence was used as the reference. (D) *p14ARF* promoter methylation in case 425 with *NBS1* mutation S638P. U: unmethylated; M: methylated; PC: methylated DNA (Zymo Research, Irvine, CA); NC: unmethylated DNA from normal blood; *M*: 50 bp DNA ladder.

Genetic alterations in the *TP53* pathway in the 82 analyzed cases of primary liver cancer are summarized in Table 3. *TP53* mutations were identified in 13.4% cases (11/82), including one frameshift mutation (616del1ins14), one stop mutation (G298X), nine missense point mutations (V157P, P301L, Q192H, R248G, R249S, E285K, R273C, R286V and Y220C) (Figure 4A). All except the frameshift mutation are known *TP53* mutations registered in the International Agency for Research on Cancer TP53 Database (R15 release, http://www-p53.iarc.fr). No case had more than one *TP53* mutation. *MDM2* amplification, *p14ARF* homozygous deletion and *p14ARF* promoter methylation were identified in five (6.1%), seven (8.5%) and 25 (30.5%) cases, respectively, and 45 (54.9%) tumors contained at least one genetic alteration in the *TP53* pathway (Figure 4B-D).

**Table 3:** Association between genetic alterations in the *TP53* pathway and *NBS1* mutation in HCC and ICC.

	HCC (*n*=64)			ICC (*n*=18)			PLC* (*n*=82)		
*NBS1* mutation	Yes	No	*P*-value**^*a*^**	Yes	No	*P*-value**^*a*^**	Yes	No	*P*-value**^*a*^**
*TP53* mutation									
Yes	2	7	0.1957	1	1	0.2157	3	8	0.0701
No	4	51		1	15		5	66	
*MDM2 amplification*									
Yes	1	3	0.3322	0	1	1.0000	1	4	0.4096
No	5	55		2	15		7	70	
*p14 homozygous* deletion									
Yes	1	5	1.0000	0	1	1.0000	1	6	1.0000
No	5	53		2	15		7	68	
*p14 promoter* methylation									
Yes	2	18	1.0000	2	3	0.0653	4	21	0.2389
No	4	40		0	13		4	53	
At least one of the above four alterations									
Yes	5	33	0.3875	2	5	0.1373	7	38	0.0672
No	1	25		0	11		1	36	

***^a^***Fisher’s exact test. ^*^PLC: primary liver cancer.

Of the eight tumor cases with *NBS1* mutations, seven (87.5%) had at least one concomitant genetic alteration in the *TP53* pathway (*P* =0.0672, table 3) and three carried *TP53* mutations (*P* =0.0701, table 3). Notably, of the five cases of tumor with the five *NBS1* mutations which are predicted to be damaging to Nbs1 function, three carried *TP53* mutations (*P* =0.0159). 

## Discussion

Many genetic and epigenetic changes have been identified in precancerous hepatic lesions and in HCC, including chromosomal amplification, mutations, loss of heterozygosity and global DNA hypermethylation [3,22]. The Wnt/β-catenin pathway is commonly disrupted in HCC, usually as a result of mutations in *CTNNB1* or AXIN1, or epigenetic silencing of *CDH1* [22]. The p53 and Rb1 pathways are often disturbed in HCC, and somatic mutations in *TP53* has been reported with the rate of 14–35% worldwide depending on the level of aflatoxin exposure [23,24].The PI3K/Akt/mTOR pathway is also commonly disrupted, sometimes due to abnormal inactivation of tyrosine kinase receptors or as a result of constitutive activation of *PI3K* following loss of function of the tumor-suppressor gene *PTEN* [25]. Derangements of other signal transduction pathways, such as the *MAPK* pathway and the *TGF-β* pathway, also play roles in hepatocarcinogenesis [23]. Therefore, HCC is characterized by remarkable molecular heterogeneity. In the present study, we provide the first evidence that mutational inactivation of *NBS1*, a DNA repair-associated gene, are involved in the pathogenesis of primary liver cancer. 

 The main functional domains of Nbs1 comprise the forkhead-associated (FHA) domain (amino acids 24–100), the breast cancer C-terminal (BRCT) domain (amino acids 105–190), the second BRCT domain (amino acids 215–324), and the Mre11-binding domain (amino acids 601–700, binding sites 665–693), as well as ATM phosphorylation sites [13,14]. Both the FHA and BRCT domains are essential for responses to DNA damage, including the formation of ionizing radiation-induced foci, S-phase checkpoint activation and nuclear focus formation after irradiation, and play crucial roles in cell survival after radiotherapy [26]. The Mre11-binding domain is essential for the formation of the MRN complex, which plays an important role in DNA damage-induced checkpoint control and DNA repair [7]. In the present study, two *NBS1* miscoding mutations were located in the FHA domain; one *NBS1* miscoding mutations was located in the second BRCT domain; three *NBS1* mutations were located close to the ATM phosphorylation sites; notably, three mutations were located in the Mre11-binding domain (Figure 2). Heterozygous *NBS1* mutations causing loss of the Mre11-binding domain predispose carriers to common types of cancer [27,28]. Thus, the *NBS1* mutations identified in the present study are likely to play significant roles in the pathogenesis of primary liver cancer.

**Figure 5:**
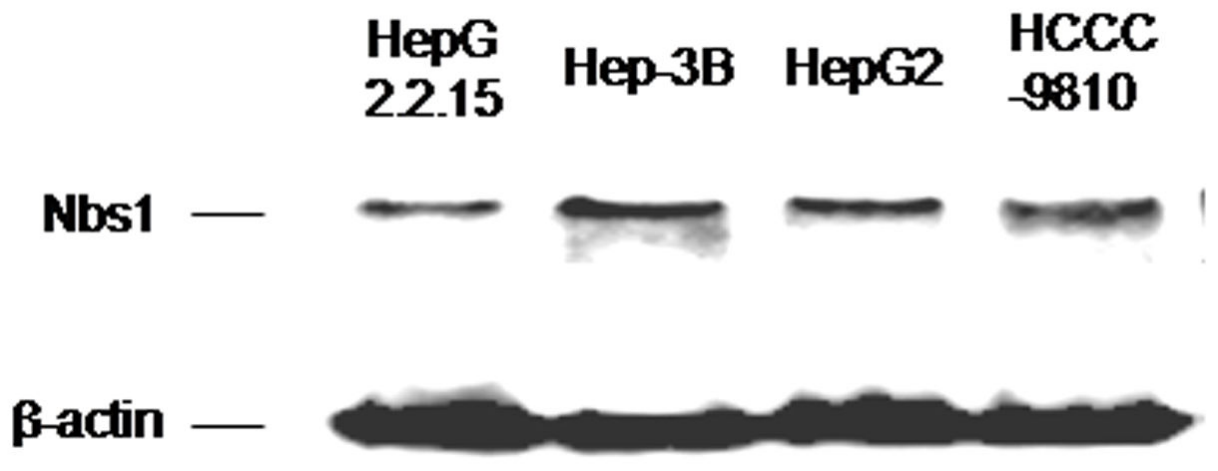
Endogenous expression of Nbs1 in HCC or ICC cell lines. Western blots analysis of endogenous expression of Nbs1 in three HCC cell lines (HepG2, Hep-3B and HepG2.2.15 ) and one ICC cell line(HCCC-9810).β-actin was used as protein loading control.

Several studies have shown that somatic heterozygous *NBS1* mutations in sporadic tumors have functional consequences for the Nbs1 protein [11,27,29]. Heterozygous *NBS1* splicing mutation (IVS11+2insT) detected in sporadic gastric, colorectal and lung cancers led to defects in crucial binding to Mre11, Mdc1 and BRCA1, and caused impaired ATM phosphorylation in response to low-dose irradiation in the heterozygous state [27]. In breast cancer cells with a *NBS1* missense mutation (R215W), levels of radiation-induced phosphorylation of Nbs1 (Ser343) were reduced to 60% of wild-type [11]. Missense *NBS1* mutations (L150F and I171V) were associated with chromosomal instability in sporadic breast cancer [29]. In the present study, three of the eight *NBS1* mutations (F603L, S638P and S633T) are located in the Mre11-binding domain of Nbs1. Since the disruption of the Mre11 binding domain of Nbs1 may lead to loss of Mre11 nuclear localization and increased staining for Mre11 in the cytoplasm [21], we were able to analyze the functional consequences of the three *NBS1* mutations located in the Mre11-binding domain by the analysis of nuclear localization of Mre11 in tumor cells. Loss of the nuclear localization of Mre11, one of the hallmarks for Nbs1 deficiency, was observed in two tumors with the *NBS1* mutation S638P or S633T, suggesting an alteration of the function of the MRN complex, consistent with previous reports that loss of Nbs1 protein from the Mre11-binding domain disrupts the nuclear localization of Mre11 and lead to deficiency in DNA DSBs repair [21,27]. The role of the mutated Nbs1 protein on HCC progression is important to evaluate its potential functional consequence on tumorigenesis. However, *NBS1* is a “caretaker” gene, which do not directly promote tumor development when inactivated. Various expression levels of Nbs1 have been detected in three HCC cell lines (HepG2, Hep-3B and HepG2.2.15) and one ICC cell line HCCC-9810 (Figure 5), and notably, Nbs1 null mutant cell lines is not viable due to the functional importance of MRN complex in cells. If we express exogenous mutant Nbs1 in HCC cells, we cannot rule out the possible role of endogenous Nbs1 in the cells. Thus, it is hard to conclude the role of *NBS1* mutations in HCC progression by construction of the *NBS1* mutation vectors and express mutated Nbs1 in HCC cell lines. It is noteworthy that, increased HCC incidence in heterozygote *NBS1* mutant mice provided a strong genetic evidence for the role of *NBS1* mutations in HCC [18].

Several studies have demonstrated a possible cooperation of loss of *TP53* and *NBS1* in the pathogenesis of neoplasms [13,14,16,17,30]. In addition to its role in DNA double-strand break repair, Nbs1 directly binds to Mdm2, a negative regulator of p53, and co-localizes to sites of DNA damage caused by gamma-irradiation, while Mdm2 over-expression inhibited DNA double-strand break repair associated with Nbs1 [16]. Using a humanized p53 knock-in allele in mice, Song et al. demonstrated that the common p53 mutants disrupt the earliest stage of DNA double-strand break damage response by interfering with recruitment of the MRN complex to the site of DNA damage, leading to inactivation of ATM and genetic instability [30]. In *NBS1* mutant/mutant mice expressing N-terminally truncated Nbs1, p53 deficiency greatly facilitated the development of thymic lymphomas [17]. Moreover, mutational inactivation of the *NBS1* gene in tumors is associated with *TP53* mutations in sporadic medulloblastoma and primary glioblastoma [13,14]. On the other hand, it was recently reported that Nbs1 regulates a novel p53 independent apoptotic pathway through *BAX* activation in response to DNA damage and that DNA damage-induced apoptosis is significantly reduced in Nbs1-deficient cells regardless of their p53 status [31]. However, the present study notably showed the co-presence of *NBS1* mutations with genetic alterations in the *TP53* pathway, implying a synergistic effect of Nbs1 disruption and p53 inactivation in liver cancer development. Although the relationship between *NBS1* mutations and *TP53* pathway alterations is not statistical significant, probably due to a limited number of tumor cases analyzed, our data has provided a potential possibility for such relationship, with the p-value around the threshold, and with statistic significant result (p=0.0159) when cases with the *NBS1* mutations which are predicted to be damaging to Nbs1 function were analyzed. 

In summary, this study has revealed the mutation inactivation of *NBS1*, a DNA repair associated gene, in HCC and ICC. These findings would be helpful in increasing understanding of the molecular pathogenesis of primary liver cancer.

## Supporting Information

Figure S1 **Nbs1 expression and p-Nbs1 level in tumor cells with or without *NBS1* mutation.** (A) Western blot analysis for detection of Nbs1 expression and p-Nbs1 level in five representative tumor cases: cases 478 (HCC) and 425 (ICC) with *NBS1* mutation D272N and S638P, Cases 327,333 and 343 without *NBS1* mutation, β-actin was used as the reference.(B) Representative IHC of Nbs1 expression and p-Nbs1 level in tumor tissue of one HCC case (case 478). Original magnification: ×40.
(TIF)

Table S1 **Association between *NBS1* mutations and clinical parameters in 64 cases of HCC.**
(DOC)

## References

[1] Theise ND, Curado MP, Franceschi S, Hytiroglou P, Kudo M et al. (2010) Hepatocellular carcinoma. In: Bosman FT Carneiro F Hruban RH Theise ND WHO Classification of Tumors of the Digestive. System: 205-216.

[2] Nakanuma Y, Curado MP, Franceschi S, Gores G, Paradis V et al. (2010) Intrahepatic cholangiocarcinoma. In: Bosman FT Carneiro F Hruban RH Theise ND WHO Classification of Tumors of the Digestive. System: 217-224.

[3] Thorgeirsson SS, Grisham TW (2002) Molecular pathogenesis of human hepatocellular carcinoma. Nat Genet 31: 339-346. doi:10.1038/ng0802-339. PubMed: 12149612. 12149612

[4] Paterlini-Bréchot P, Saigo K, Murakami Y, Chami M, Gozuacik D et al. (2003) Hepatitis B virus related insertional mutagenesis occurs frequently in human liver cancers and recurrently targets human telomerase gene. Oncogene 22: 3911-3916. doi:10.1038/sj.onc.1206492. PubMed: 12813464. 12813464

[5] Zhu DQ, Huang ZY (2007) DNA damage and Iiver cancer. World Chinese Journal of Digestology.15: 1775-1780.

[6] Stracker TH, Theunissen JW, Morales M, Petrini JH (2004) The MRE11 complex and the metabolism of chromosome breaks: the importance of communicating and holding things together. DNA Repair (Amst) 3: 845-854. doi:10.1016/j.dnarep.2004.03.014. PubMed: 15279769. 15279769

[7] Bill CA, Summers T (2004) Genomic DNA double-strand breaks are targets for hepadnaviral DNA integration. Proc Natl Acad Sci of the USA 101: 11135-11140. doi:10.1073/pnas.0403925101. PubMed: 15258290. PMC503752 15258290

[8] Dandri M, Burda MR, Bürkle A, Zuckerman DM, Will H et al. (2002) Increase in de novo HBV DNA integrations in response to oxidative DNA damage or inhibition of poly(ADPribosyl) ation. Hepatology 35: 217-223. doi:10.1053/jhep.2002.30203. PubMed: 11786979. 11786979

[9] Varon R, Seemanova E, Chrzanowska K, Hnateyko O, Piekutowska-Abramczuk D et al. (2000) Clinical ascertainment of Nijmegen breakage syndrome (NBS) and prevalence of the major mutation, 657del5, in three Slav populations. Eur J Hum Genet 8: 900-902. doi:10.1038/sj.ejhg.5200554. PubMed: 11093281. 11093281

[10] Lee JH, Lim DS (2006) Dual role of NBS1 in the ataxia telangiectasia mutated-dependent DNA damage response．FEBS J 273: 1630-1636. doi:10.1111/j.1742-4658.2006.05191.x. PubMed: 16623700. 16623700

[11] Bogdanova N, Feshchenko S, Schürmann P, Waltes R, Wieland B et al. (2008) Nijmegen breakage syndrome mutations and risk of breast cancer. Int J Cancer 122: 802-806. doi:10.1002/ijc.23168. PubMed: 17957789. 17957789

[12] Nowak J, Mosor M, Ziółkowska I, Wierzbicka M, Pernak-Schwarz M et al. (2008) Heterozygous carriers of the I171V mutation of the NBS1 gene have a significantly increased risk of solid malignant tumors. Eur J Cancer 44: 627-630. doi:10.1016/j.ejca.2008.01.006. PubMed: 18280732. 18280732

[13] Huang J, Grotzer MA, Watanabe T, Hewer E, Pietsch T et al. (2008) Mutations in the Nijmegen breakage syndrome gene in medulloblastomas. Clin Cancer Res 14: 4053-4058. doi:10.1158/1078-0432.CCR-08-0098. PubMed: 18593981. 18593981

[14] Watanabe T, Nobusawa S, Lu SQ, Huang J, Mittelbronn M et al. (2009) Mutational inactivation of the Nijmegen breakage syndrome gene (NBS1) in glioblastomas is associated with multiple TP53 mutations. J Neuropathol Exp Neurol 68: 210-215. doi:10.1097/NEN.0b013e31819724c2. PubMed: 19151620. 19151620

[15] Chrzanowska KH, Piekutowska-Abramczuk D, Popowska E, Gładkowska-Dura M, Małdyk J et al. (2006) Carrier frequency of mutation 657del5 in the NBS1 gene in a population of Polish pediatric patients with sporadic lymphoid malignancies.Int J Cancer 118: 1269-1274. doi:10.1002/ijc.21439. PubMed: 16152606. 16152606

[16] Alt JR, Bouska A, Fernandez MR, Cerny RL, Xiao H et al. (2005) Mdm2 binds to NBS1 at sites of DNA damage and regulates double strand break repair. J Biol Chem 280: 18771-18781. doi:10.1074/jbc.M413387200. PubMed: 15734743. 15734743

[17] Kang J, Ferguson D, Song H, Bassing C, Eckersdorff M et al. (2005) Functional interaction of H2AX, NBS1, and p53 in ATM-dependent DNA damage responses and tumor suppression. Mol Cell Biol 25: 661-670. doi:10.1128/MCB.25.2.661-670.2005. PubMed: 15632067. 15632067 PMC543410

[18] Dumon-Jones V, Frappart PO, Tong WM, Sajithlal G, Hulla W et al. (2003) Mice heterozygous for NBN (the murine homologue of NBS1) developed a wide variety of tumors affecting the liver, mammary gland, prostate, and lung, in addition to lymphomas. Cancer Res 63: 7263-7269. PubMed: 14612522.14612522

[19] Huang J, Zhao YP, Li Q, Zhang JX, Wang Y et al. (2012) Association of single nucleotide polymorphisms of NBS1 gene with genetic susceptibility to primary liver cancer in a Chinese Han population. Prog Biochem Biophys 39: 678-686. doi:10.3724/SP.J.1206.2011.00536.

[20] Di YM, Chan E, Wei MQ, Liu JP, Zhou SF (2009) Prediction of Deleterious Non-synonymous Single-Nucleotide Polymorphisms of Human Uridine Diphosphate Glucuronosyltransferase Genes. AAPS J 11: 469-480. doi:10.1208/s12248-009-9126-z. PubMed: 19572200. 19572200 PMC2758119

[21] Yang YG, Frappart PO, Frappart L, Wang ZQ, Tong WM (2006) A novel function of DNA repair molecule NBS1 in terminal differentiation of the lens fibre cells and cataractogenesis. DNA Repair (Amst) 5: 885-893. doi:10.1016/j.dnarep.2006.05.004. PubMed: 16790366. 16790366

[22] Mínguez B, Tovar V, Chiang D, Villanueva A, Llovet JM (2009) Pathogenesis of hepatocellular carcinoma and molecular therapies. Curr Opin Gastroenterol 25: 186-194. doi:10.1097/MOG.0b013e32832962a1. PubMed: 19387255. 19387255

[23] Aravalli RN, Steer CJ, Cressman EN (2008) Molecular mechanism of hepatocellular carcinoma. Hepatology 48: 2047-2063. doi:10.1002/hep.22580. PubMed: 19003900. 19003900

[24] Ozturk M (1991) p53 mutation in hepatocellular carcinoma after aflatoxin exposure. Lancet 338: 1356-1359. doi:10.1016/0140-6736(91)92236-U. PubMed: 1682737. 1682737

[25] Villanueva RR, Chiang DY, Newell P, Peix J, Thung S et al. (2008) Pivotal role of mTOR signaling in hepatocellular carcinoma. Gastroenterology 135: 1972-1983. doi:10.1053/j.gastro.2008.08.008. PubMed: 18929564. 18929564 PMC2678688

[26] Zhao S, Renthal W, Lee EY (2002) Functional analysis of FHA and BRCT domains of NBS1 in chromatin association and DNA damage responses. Nucleic Acids Res 30: 4815-4822. doi:10.1093/nar/gkf612. PubMed: 12433983. 12433983 PMC137160

[27] Ebi H , Matsuo K, Sugito N, Suzuki M, Osada H et al. (2007) Novel NBS1 heterozygous germ line mutation causing MRE11-binding domain loss predisposes to common types of cancer. Cancer Res 67: 11158-11165. doi:10.1158/0008-5472.CAN-07-1749. PubMed: 18056440. 18056440

[28] Desai-Mehta A, Cerosaletti KM, Concannon P (2001) Distinct functional domains of nibrin mediate MRE11 binding, focus formation, and nuclear localization. Mol Cell Biol 21: 2184-2191. doi:10.1128/MCB.21.6.2184-2191.2001. PubMed: 11238951. 11238951 PMC86852

[29] Heikkinen K, Rapakko K, Karppinen SM, Erkko H, Knuutila S et al. (2006) RAD50 and NBS1 are breast cancer susceptibility genes associated with genomic instability. Carcinogenesis 27: 1593-1599. PubMed: 16474176. 16474176 10.1093/carcin/bgi360 PMC3006189

[30] Song H, Hollstein M, Xu Y (2007) p53 gain-of-function cancer mutants induce genetic instability by inactivating ATM. Nat Cell Biol 9: 573-580. doi:10.1038/ncb1571. PubMed: 17417627. 17417627

[31] Iijima K, Muranaka C, Kobayashi JY (2008) NBS1 regulates a novel apoptotic pathway through Bax activation. DNA Repair (Amst) 7: 1705-1716. doi:10.1016/j.dnarep.2008.06.013. PubMed: 18644472. 18644472

