# Supplementary figures and images for: Mutation Inactivation of Nijmegen Breakage Syndrome Gene (NBS1) in Hepatocellular Carcinoma and Intrahepatic Cholangiocarcinoma
Source: PLoS One. 2013 Dec 13;8(12):e82426. doi: 10.1371/journal.pone.0082426 (PMC3862623; doi:10.1371/journal.pone.0082426)

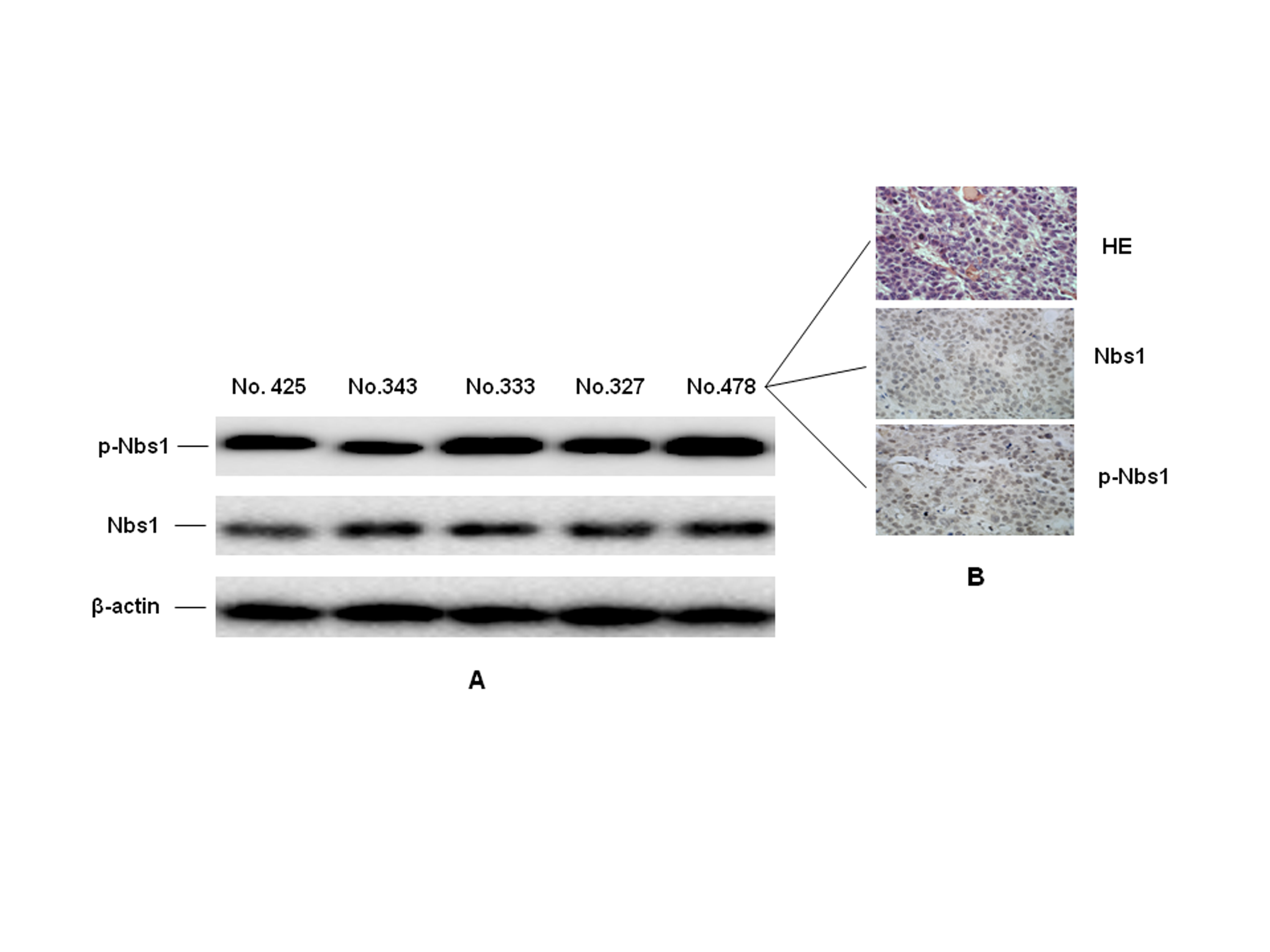

Supplement: Figure S1 — Nbs1 expression and p-Nbs1 level in tumor cells with or without NBS1 mutation. (A) Western blot analysis for detection of Nbs1 expression and p-Nbs1 level in five representative tumor cases: cases 478 (HCC) and 425 (ICC) with NBS1 mutation D272N and S638P, Cases 327,333 and 343 without NBS1 mutation, β-actin was used as the reference.(B) Representative IHC of Nbs1 expression and p-Nbs1 level in tumor tissue of one HCC case (case 478). Original magnification: ×40. (TIF) [file pone.0082426.s001.tif]
