# Supplementary material for: Mutation Inactivation of Nijmegen Breakage Syndrome Gene (NBS1) in Hepatocellular Carcinoma and Intrahepatic Cholangiocarcinoma
Source: PLoS One. 2013 Dec 13;8(12):e82426. doi: 10.1371/journal.pone.0082426 (PMC3862623; doi:10.1371/journal.pone.0082426)
Supplement: Table S1 — Association between NBS1 mutations and clinical parameters in 64 cases of HCC. (DOC) [file pone.0082426.s002.doc]

**Table S1. Association between *NBS1* mutations and clinical parameters in 64 cases of HCC**

| Clinical parameter | *NBS1* mutation | | *P*-value^a^ |
| --- | --- | --- | --- |
|  | + | – |  |
| Tumor stage |  |  |  |
| Stage 1 | 4 | 34 | 1.0000 |
| >Stage 1 | 2 | 24 |  |
| HBV infection |  |  |  |
| Positive | 5 | 47 | 1.0000 |
| Negative | 1 | 11 |  |
| Differentiation |  |  |  |
| Well or moderate | 6 | 42 | 0.3231 |
| Poor | 0 | 16 |  |

^a^Fisher’s exact test.
